# Cardiovascular health: an important component of cancer survivorship

**DOI:** 10.1136/bmjonc-2023-000090

**Published:** 2023-10-13

**Authors:** Siobhan Cleary, Stuart D Rosen, Duncan C Gilbert, Ruth E Langley

**Affiliations:** 1 MRC Clinical Trials Unit at University College London, Institute of Clinical Trials and Methodology, London, UK; 2 Department of Cancer and Surgery, Imperial College Healthcare NHS Trust, London, UK; 3 National Heart and Lung Institute, Imperial College London, London, UK

**Keywords:** Neoplasms

## Abstract

Advances in the detection and treatment of cancer have translated into improved cancer survival rates and a growing population of cancer survivors. These include those living with cancer and individuals free of the disease following treatment. Epidemiological studies demonstrate that cancer survivors are at an increased risk of cardiovascular disease (CVD), with cardiovascular (CV) mortality overtaking cancer mortality in some tumour types. Cancer and CVD share common aetiological risk factors, for example, age, tobacco use and obesity, as well as a shared inflammatory pathogenesis. The CV risks of mediastinal radiotherapy and chemotherapy, first observed in the 1970s with anthracyclines, have long been appreciated. More recently, targeted anticancer therapeutics (human epidermal growth factor receptor-2 targeted therpies, vascular endothelial growth factor inhibitors, second/third-generation BCR-ABL inhibitors, multiple myeloma therapies and combination RAF and MEK inhibitors in particular) as well as immunotherapies have added to the burden of treatment-related CV toxicity. Additionally, cancer therapy may indirectly impact on CV health by decreasing physical activity, increasing weight gain and accelerating the ageing process. Improving overall health outcomes by considering cardiological prevention and management in cancer survivorship is an area of increasing interest. CV risk factor assessment and management are recommended post-cancer treatment in accordance with primary prevention guidelines. The European Society of Cardiology 2022 guidelines also recommend enhanced surveillance after cancer treatments with a moderate to high risk of CV consequences. The aim of this article is to provide an overview of the interconnections between cancer and CVD, review current survivorship recommendations, and highlight key areas of ongoing and future research.

## Introduction

Advances in the detection and treatment of cancer over recent decades have translated into substantial improvements in cancer survival rates. In high-income countries, cancer survival has doubled in the past 40 years, with half of those diagnosed now expected to live for at least 10 years.^1^ This trend, coupled with the rise in incident cancers, driven mainly by ageing populations, means there are an increasing number of cancer survivors.^2 3^ Cancer survivors include those living with cancer, as well as individuals free of the disease following treatment; it is estimated that there will be over 26 million cancer survivors in the USA by 2040.^2^

Longitudinal studies of childhood and young adult cancer survivors have long observed significant rates of chronic morbidity in long-term survivors, including elevated cardiovascular (CV) risk and premature mortality, with CV disease (CVD) the leading cause of premature non-cancer mortality.^4–6^ While the substantial differences between paediatric and adult cancer survivors preclude generalisability of this observation, a growing number of studies have highlighted elevated rates of CV morbidity and mortality in adult cancer survivors.^7–12^ Cancer shares aetiological risk factors with CVD, in particular age, tobacco consumption and obesity, as well as common underlying pathogeneses. Cancer treatments may also increase CV risk in multiple ways.

Data from the US Surveillance, Epidemiology and End Results (SEER) Database including 7.5 million cancer survivors show a twofold increase in risk of fatal heart disease compared with the background population (standardised mortality rate (SMR) 2.24; 95% CI 2.23 to 2.25, relative risk (RR) p<0.0001).^7^ Increased risk was seen in all 12 tumour types studied but varied in magnitude. The highest risk was seen with lung cancer and myeloma where the SMR was 7–14 (RR p<0.0001) in the first year and remained at 4–5 after 10 years. Across tumour types, following an early peak in CV mortality attributed to acute treatment effects, risk declined but remained elevated in comparison with the background population before again increasing over time, with an overall SMR after 10 years of 2.73 (95% CI: 2.7 to 2.75; RR p<0.0001).^7^ Similarly, in an English population-based cohort study of 108 215 cancer survivors across 20 tumour types, increased risks of venous thromboembolism (VTE), heart failure, cardiomyopathy, arrhythmia, pericarditis, coronary artery disease and valvular heart disease were seen and varied substantially according to tumour type.^8^ Younger age at diagnosis was associated with greater relative increase in risk, though more deaths were seen in older age groups.^8^ While conventional CV risk factors were slightly more prevalent in cancer survivors, exploratory analyses suggested these did not drive the increased CV morbidity, although increased risk was associated with chemotherapy use.^8^

A recent study in the UK biobank of 18 714 participants with a previous cancer diagnosis compared with matched non-cancer controls found cancer was independently associated with a range of CVDs.^9^ Furthermore, in a subset of 1354 participants who underwent cardiac magnetic resonance, cancer history was predictive of adverse cardiac remodelling independent of traditional vascular risk factors.^9^ Conversely, a retrospective cohort study of 36 232 2-year cancer survivors without CVD indicated the importance of traditional vascular risk factors, including hypertension, diabetes and dyslipidaemia, which were significantly higher than in non-cancer controls and predictors of developing CVD.^10^ This study also highlighted the detrimental consequence of a diagnosis of CVD; the 8-year overall survival (OS) for cancer survivors diagnosed with CVD was 60% compared with 81% for those without CVD.^10^

Premature CVD morbidity and mortality may be of particular importance in long-term cancer survivors, where studies suggest CV mortality may compete with or surpass that of the index cancer. In a SEER Database study of 3 234 256 US cancer survivors, including 28 tumour types, 38% of participants died from cancer and 11.3% from CVDs, 76.3% of which were due to heart disease.^11^ The highest rates of CV deaths were seen in those with cancer of the urinary bladder (19.4%), larynx (17.3%), prostate (16.6%), uterus (15.6%), colorectum (13.7%) and breast (11.7%).^11^ This highlights the commonality of CV deaths in cancer survivors, including in several common tumour types, where almost half of all CV deaths for the whole cohort occurred in survivors of breast or prostate cancer.^11^ SMRs demonstrated elevated CV mortality in cancer survivors compared with the general population, but additional analyses examined the differential risk of CV deaths according to tumour prognosis where for 11 tumour types, including breast and prostate cancer, the risk of cancer death differed by <10%, was equal to or even surpassed by the risk of CV death.^11^ This study again noted an acute peak in CV mortality in the year of cancer diagnosis, possibly a reflection of the interplay between treatment factors, pre-existing CV risk and tumour burden, followed by a chronic phase of persisting elevated risk which increased over time, where late treatment impacts or accelerated pathology may contribute.^13^ In another study of 104 028 English 1-year cancer survivors, following a diagnosis of one of nine common cancers, CV mortality overtook cancer mortality in all tumour types for those aged ≥80 years between 2 and 11 years after diagnosis and in seven tumour types for those aged 60–79 years after 5–17 years.^12^

These studies highlight the importance of considering the comorbid health of cancer survivors, and the long-term risks faced by this group. Given the potential impacts of premature CV morbidity and mortality on survival and quality of life in cancer survivors, understanding key drivers of risk and developing integrated survivorship strategies are areas of increasing interest and value.^14^

### Shared risk factors

#### Inherent risk factors

The prevalence of CVD and cancer are both strongly linked to increasing age. Cancer incidence increases sharply after the age of 50 years, with 90% of incident cancers occurring in that group.^15 16^ Similarly, CVD primarily affects those over 50 years.^17^ A number of the biological hallmarks of ageing including DNA damage, cellular senescence, epigenetic alterations and telomere attrition are shared with cancer.^18^ This interconnection is illustrated in cancer survivors who have an excess of ageing phenotypes, including frailty, sarcopenia, cognitive decline, secondary cancers and CVD.^19 20^

Shared genetic factors have also been associated with elevated risk of CVD and cancer, including mutations of the JAK2, TTN, TET2 and ATM genes with gene network analyses highlighting links with DNA damage response pathways including homologous recombination.^21^ Mutations in genes including TET2 are also implicated in clonal haematopoiesis of indeterminate potential (CHIP). CHIP is strongly linked with ageing, increased risk of haematological malignancies and CVD.^22^

#### Modifiable risks

Tobacco exposure remains the leading modifiable risk factor for cancer and is an important risk factor for CVD, accounting for an estimated 2.5 million cancer deaths and 3.2 million CVD deaths globally in 2019.^23 24^ Although overall rates of cigarette smoking have declined, substantial geographical variations remain with an estimated 80% of current smokers residing within low-income and middle-income countries where cancer incidence is increasing.^23^ Proatherogenic consequences include abnormal lipid oxidation, endothelial dysfunction, promotion of inflammation and a prothrombotic tendency.^25^ Genetic mutations resulting from inhaled carcinogens are central to tobacco-related tumourigenesis, with associated inflammation and oxidative stress also tumour promoting.^26^

Diet, obesity and a lack of physical exercise are interlinked and associated with increased risk of both diseases. Dietary patterns and nutrition have strong associations with CV risk, primarily mediated through effects on adiposity, hypertension, dyslipidaemia and insulin resistance.^27^ Reduced colorectal cancer incidence is associated with the consumption of whole grains, dietary fibre and dairy products, while increased risk is associated with red and processed meat.^28^ The Mediterranean diet recommended for the prevention of CVD has been associated with a reduced risk of cancer, although this could be confounded by increased levels of physical activity in its adherents.^28^

Global trends in body mass index (BMI) show year-on-year rises with over half the adult population now estimated to be either overweight (BMI ≥ 25) or obese (BMI ≥ 30).^29 30^ Obesity-related deaths have more than doubled since 1990, two-thirds of which are due to CVD.^29 31^ Excess body fat is associated with an increased risk of developing 13 cancers and accounted for an estimated 4.6% of cancer deaths in 2019.^32 33^ Obesity’s association with CVD is mediated through several mechanisms including elevated blood pressure, dyslipidaemia and insulin resistance.^34^ Its role in carcinogenesis is thought to be due to hormonal changes, insulin resistance, disruption to insulin-like growth factor pathways and chronic subclinical inflammation.^35^ Similar biological pathways may also explain the association between diabetes mellitus and an elevated risk of several cancers.^36^ Diabetic complications also include a twofold increase in atherosclerotic CVD.^37 38^

Sufficient levels of physical activity are associated with broad health benefits including reduced incidences of CVD and certain cancers; however, levels of physical inactivity are rising.^39 40^ Global estimates suggest around 6% of CVD and 10% of breast and colon cancers are the result of insufficient physical activity.^39^ Physical activity modulates several traditional CV risk factors including blood pressure, lipid profiles, insulin sensitivity and adiposity.^41^ However, benefits in incident CVD and cancers are observed independent of traditional risk factors reflecting the multimodality biological impacts of physical activity. Exercise also reduces prothrombotic tendency and systemic inflammation, improves autonomic regulation and vascular endothelial function, and alleviates age-related declines in muscle strength and function.^41 42^ Biological pathways hypothesised to influence cancer risk associated with levels of physical activity and adiposity include modulation of metabolic pathways, insulin sensitivity, chronic inflammation and endogenous sex hormones.^35^ A number of additional pathways including oxidative stress, epigenetic alterations, immune function and the microbiome have also been proposed.^35^

Indirect consequences of anticancer treatment such as deconditioning, weight gain and physical inactivity may further increase the CV risk in cancer survivors. Weight gain and physical inactivity appear to increase following cancer treatment, with population trends showing obesity has risen faster in cancer survivors than the background population.^43^ Studies of cardiorespiratory fitness (CRF) show evidence of post-treatment declines across the cancer survivorship continuum, including an estimated 30% decline in women undergoing primary adjuvant therapy for breast cancer.^44^ CRF is a global measure integrating the ability of the CV system to transport oxygen to skeletal muscle during exercise, and clinical evidence supports an independent and graded association between CRF and both CV and all-cause mortality in adult populations.^45 46^ An inverse relationship has also been observed between CRF and incident cancers and mortality in cancer survivors.^47 48^ A recent study of 1632 adult cancer survivors found CRF was a strong independent predictor of all-cause, CV and cancer mortality.^49^

### Shared biological mechanisms

**Figure 1:**
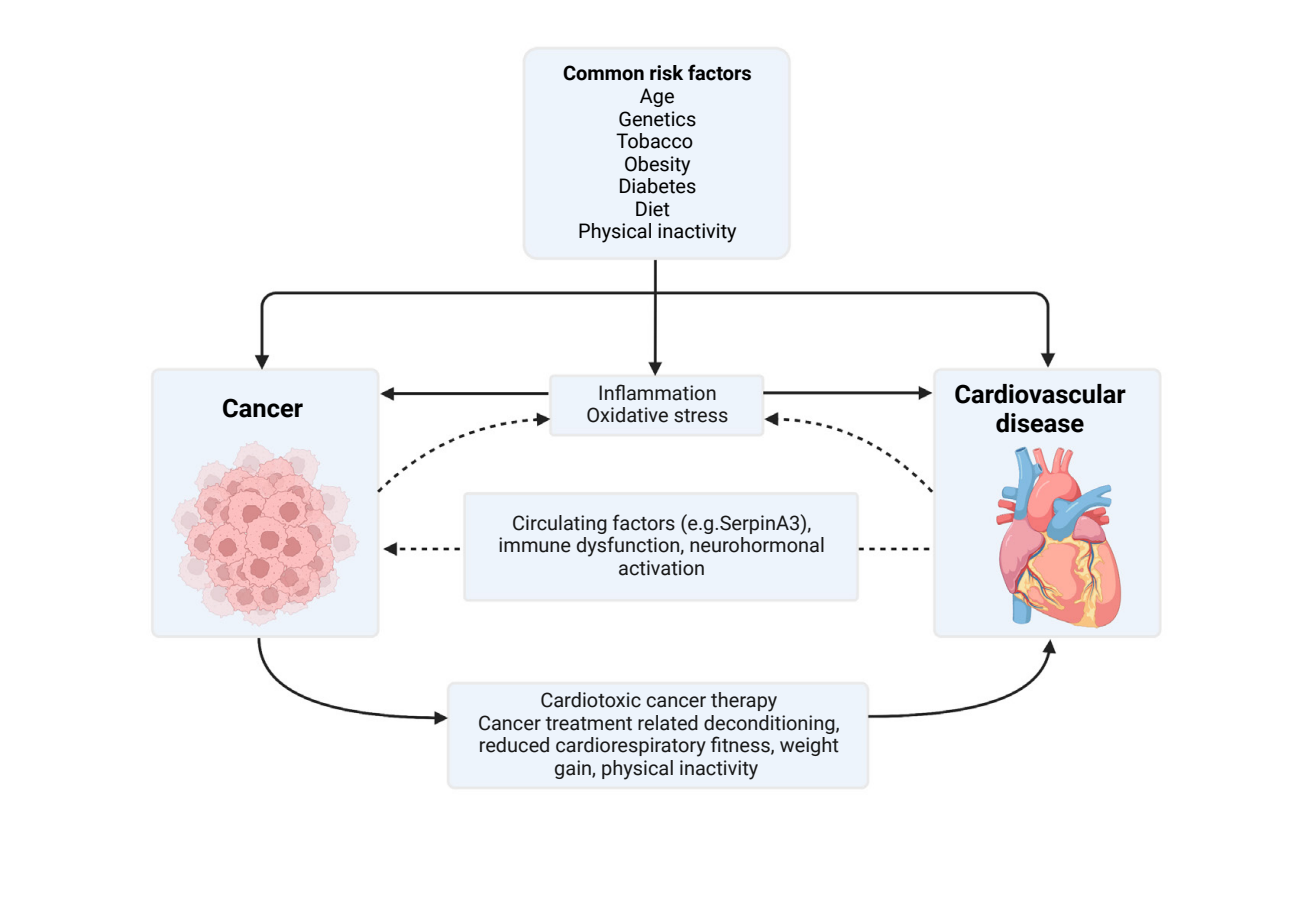
Overview of the interconnections between cancer and CVD. Dotted lines illustrate potential interconnections between pathophysiological processes which may contribute to a bidirectional relationship between cancer and CVD. Created with Biorender.com. CVD, cardiovascular disease.

Insights into the biology of cancer and CVD suggest several shared pathological mechanisms, including inflammation and oxidative stress, with emerging evidence of a possible bidirectional relationship (figure 1).^50^ Oxidative modification of lipids, with ensuing endothelial dysfunction and increased leucocyte–endothelial adhesion, is a key early step in atheroma development.^51^ Alterations in cell signalling, DNA damage and upregulation of inflammatory pathways in the presence of oxidative stress also appear to promote tumourigenesis and influence cancer evolution.^52^ Chronic inflammation may promote tumourigenesis via modulation of several important pathways including provision of growth stimuli, transcriptional change, epigenetic alterations and epithelial to mesenchymal transition.^53^ Furthermore, a growing body of evidence suggests complex platelet–tumour interactions assist in producing a favourable environment for tumour progression, invasion and metastasis.^54^ In vivo markers of platelet activation have also been associated with all-cause, CV and cancer mortality.^55^

Over the past two decades, inflammation has been implicated at each step of atherogenesis, although the causal link has been debated.^56 57^ Inflammatory mediators are elevated in heart failure, and C reactive protein (CRP) is associated with increased risk of CV events and the development of heart failure.^58–60^ Clinical evidence that reducing inflammation can reduce CV events was reported in the CANTOS trial (NCT01327846). Canakinumab, a monoclonal antibody against interleukin-1β, at 300 mg, 150 mg and 50 mg, was compared with placebo in 10 061 participants with a history of myocardial infarction and elevated high-sensitivity CRP. Interestingly, only the 150 mg evaluation met the prespecified significance threshold for reducing CV events (hazard ratio (HR) 0.85, 95% CI: 0.74 to 0.98; p=0.021).^61^ Canakinumab use was, however, associated with an increased risk of fatal infections and thrombocytopenia without any significant difference in haemorrhage risk.^61^ Exploratory analyses also found a reduced incidence of lung cancer and cancer mortality with canakinumab.^62^ However, subsequent phase III trials of canakinumab in locally advanced and metastatic non-small cell lung cancer failed to reach their primary outcome measure of prolonging OS.^63^

Several studies have observed increased incident cancers in patients with heart failure leading to suggestions of a possible bidirectional relationship.^64–68^ This has been hypothesised to relate to shared aetiologies, biology and genetics with preclinical studies suggesting heart failure itself may promote tumourigenesis.^69 70^ In addition to impacts on inflammation and oxidative stress, implicated pathophysiological pathways include cardiac and other circulating factors, immune dysfunction and neurohormonal activation.^68^ In mouse models, heart failure after large anterior myocardial infarction was associated with enhanced colonic tumour growth, possibly related to elevated cardiac mediators including serpinA3 which increases tumour growth in vitro.^71^ In a further study using transverse aortic constriction, a model for pressure overload induced heart failure, injection of cancer cells resulted in larger tumours with higher proliferation indexes and increased numbers of metastases compared with controls.^72^

Compensatory activation of the renin–angiotensin–aldosterone system occurs in heart failure to maintain blood pressure and cardiac output; however, detrimental consequences occur with chronic stimulation.^68^ In preclinical models, renin–angiotensin overactivation has been associated with increased tumour cell survival, proliferation, migration, and angiogenesis and enhanced sympathetic nervous system activity with reduced antitumoural immunity.^73 74^ In mouse models, myocardial infarction accelerated breast cancer growth with an immunosuppressive intratumoural immune landscape, suggesting altered innate immune responses in acute myocardial infarction could impact tumour development.^75^

### CV toxicities of oncological treatments

The survival benefits associated with advancing cancer therapeutics have also increased the potential for treatment-related toxicity. CV toxicities of cancer therapies include myocardial dysfunction, heart failure, ischaemic and valvular heart disease, arrhythmias, thromboembolic disease, arterial and pulmonary hypertension, and pericardial disease.^76^ Indirect CV consequences can occur due to physiological or haemodynamic effects of therapy including fluid retention and increased blood pressure.^77^ Underlying CV risk factors such as hypertension and diabetes mellitus increase the risk of treatment-related cardiotoxicity and are of particular concern in ageing populations with cancer.^10 78–81^

Chemotherapy related myocardial dysfunction is seen most prominently with anthracyclines and heart failure may develop several years after treatment. In a study of 2625 patients treated with anthracycline chemotherapy, 9% developed cardiac impairment, of which 98% had detectable declines in left ventricular ejection fraction of >10% within the first year post-treatment.^79^ Anthracycline-induced cardiac damage is generally irreversible but early detection is associated with improved outcomes underlining the importance of monitoring for subclinical toxicity.^79 81^ Other examples of chemotherapy-induced CV toxicity include acute myocardial ischaemia, either due to vasospasm such as with fluoropyrimidines or arterial thrombus best described with platinum agents, and conduction abnormalities.

In addition to acute toxicity, chemotherapy exposure has been associated with late consequences, with accelerated vascular ageing described in clinical cohorts following exposure to cisplatin and anthracyclines.^82 83^ Long-term studies of men treated for testicular cancer with cisplatin-based chemotherapy report a 1.4-fold to 7.1-fold increase in CV risk thought to be due to both direct vascular consequences of cisplatin exposure and metabolic risk factors including diabetes, hypertension and dyslipidaemia due to hypogonadism.^83–85^

In men receiving androgen deprivation therapy (ADT) for prostate cancer, observational studies highlight the increased risk of metabolic consequences, CV risk and mortality.^86^ In a population-based cohort study of over 73 196 men with locoregional prostate cancer, ADT with a luteinising hormone-releasing hormone agonist was associated with increased risk of coronary heart disease (HR 1.16; 95% CI: 1.10 to 1.21; p<0.001), myocardial infarction (HR 1.11; 95% CI: 1.01 to 1.21; p=0.03), sudden cardiac death (HR 1.16; 95% CI: 1.05 to 1.27; p=0.004) and incident diabetes (HR 1.44; 95% CI: 1.34 to 1.55; p<0.001).^87^ Excess risk of diabetes was also observed with androgen deprivation achieved through orchidectomy (HR 1.34; 95% CI: 1.20 to 1.50; p<0.001) but without significant differences in cardiac outcomes.^87^ Use of novel antiandrogen agents, such as abiraterone or enzalutamide, used in combination with ADT, also increases the risk of hypertension and CV adverse events in phase III trials.^86^ In breast cancer survivors, aromatase inhibitor (AI) use has been associated with increased risk of dyslipidaemia, hypertension, ischaemic heart disease and heart failure, compared with the selective oestrogen receptor modulator tamoxifen.^88 89^ In a meta-analysis of RCTs comparing adjuvant AIs with tamoxifen, there was a 30% increase in risk of ischaemic heart disease with AI use (RR: 1.30, 95% CI: 1.11 to 1.53).^90^ However, lower rates of myocardial infarction have been observed with tamoxifen versus placebo so whether this is due to a protective effect from tamoxifen remains uncertain.^89 90^ Consistent data highlight elevated risk of VTE with tamoxifen use, with some evidence it may increase body fat, triglycerides and risk of diabetes.^89^

Radiotherapy (RT)-associated CVD includes accelerated atherosclerosis, valvular and pericardial diseases, cardiomyopathy, conduction disease and autonomic dysfunction. Typically, this occurs late (>10 years after treatment) making estimations of the burden of RT-induced CVD difficult to quantify.^91 92^ As a locoregional therapy, CV toxicity is limited to where the radiation field includes the heart or major blood vessels, including treatment of breast, lung and oesophageal cancers, and lymphoma. In a case–control study of 2168 women who received RT for breast cancer, a linear relationship was observed between mean RT heart dose and subsequent major coronary events.^93^ Risk increased by 7.4%/Gy without an apparent threshold (95% CI: 2.9% to 14.5%; p<0.001), and remained elevated at 20 years.^93^ Recognition of these adverse consequences and advancing RT techniques has led to the development of dose/volume limits.^94^ Using data from historical studies may therefore overestimate the risk of current practice.

Recent decades have seen substantial and rapid progress in anticancer therapeutics, with targeted therapies and immunotherapies. Many of these are associated with CV toxicities and are increasingly used in potentially curative settings. Initial high rates of cardiac dysfunction were observed with the human epidermal growth factor receptor-2 (HER-2) monoclonal antibody trastuzumab when co-administered with anthracyclines in breast cancer management. Rates of CV toxicity were subsequently lowered with use of sequential regimes.^95^ In a pooled analysis of 7445 patients enrolled in three adjuvant clinical trials, asymptomatic or mildly symptomatic myocardial dysfunction with trastuzumab occurred in 8.7% and severe congestive cardiac failure in a further 2.3%.^96^ However, this may underestimate the true incidence of cardiac dysfunction, with higher rates of myocardial dysfunction and heart failure reported in real-world cohort studies which include more representative populations with cancer.^97^ Novel HER-2 targeted agents have also been developed including monoclonal antibodies, antibody–drug conjugates and tyrosine kinase inhibitors (TKIs), with safety data to date not showing additional CV risk.^95^

The vascular endothelial growth factor (VEGF) signalling pathway plays a critical role in regulation of angiogenesis. Several anticancer agents have been developed to target this pathway, including monoclonal antibodies and TKIs.^98^ Acute and dose-dependent arterial hypertension is the best characterised vascular toxicity of anti-angiogenics, with additional excess rates of arterial thromboembolism, cardiac dysfunction and ischaemia.^99 100^ In a meta-analysis of 77 phase III trials evaluating drugs targeting the VEGF pathway, hypertension occurred in 22%, severe hypertension in 7.4%, arterial thromboembolism in 1.8%, cardiac dysfunction in 2.7% and cardiac ischaemia in 1.7%, although fatal CV events were rare.^99^

TKIs of the BCR-ABL mutation are also associated with significant CV adverse events and produce off-target toxicity due to multikinase inhibition.^101^ Substantial variability exists in type and frequency of CV toxicity, ranging from the low-risk profile seen with the first-generation inhibitor imatinib, to high rates of arterial occlusive events with the third-generation TKI ponatinib.^101 102^ In a phase II trial of ponatinib, the cumulative incidence of arterial occlusive events was 25% at 5 years, 10% of which were cardiovascular, 7% cerebrovascular and 8% peripheral vascular events.^102^ Combination TKI therapy targeting BRAF and MEK pathways is also associated with significant CVD. In clinical trials, toxicities included hypertension (6–26%), left ventricular systolic dysfunction (2–12%), VTE (1–4%), QT prolongation (1–5%) and atrial fibrillation (1–4%).^103^

Prolongation of the QT interval is a risk of multiple anticancer therapies and supportive agents; thus, the assessment of baseline risk, concurrent prescribing and consideration of the required monitoring are important considerations.^104^ QT prolongation is seen with cyclin-dependent kinase 4 and 6 inhibitors used to treat breast cancer, most notably with ribociclib.^104^ In phase III trials, risk of QT prolongation with ribociclib was especially high when co-administered with tamoxifen compared with an AI.^105^ Inhibitors of the epidermal growth factor receptor are also associated with QT prolongation, with the highest risk seen with osimertinib which has additionally been linked with elevated risk of supraventricular tachyarrhythmias and heart failure.^106^

Immune checkpoint inhibitors (ICIs) have revolutionised oncological outcomes in several tumour types over the last decade. Blockage of negative co-stimulatory T cell receptors, such as PD-1 and CTLA-4, can rejuvenate antitumour T cell responses but risks immune-mediated toxicity. CV immune-mediated toxicity includes myocarditis, myocardial dysfunction, conduction disorders and acute coronary syndromes.^81 92^ While myocarditis is uncommon, with incidence estimates of between 0.04% and 1.14%, significant mortality of up to 50% of those affected has been reported.^107–109^ A matched cohort study of 2842 patients receiving ICI therapy found a threefold increased risk of CV events compared with matched controls (HR 3.3, 95% CI, 2.0–5.5; *P*<0.001) and a greater than threefold increase in aortic atherosclerotic plaque progression.^110^ In mouse models, ICIs accelerated atherosclerotic plaque development and destabilisation, characterised by increased infiltrate of cytotoxic CD8+ T cells.^111 112^ Preclinical models have also demonstrated short-term exposure to ICIs increased vascular and myocardial inflammation.^113^ The potential implications of such an impact on atherosclerosis could be significant as ICIs gain increasing indications including in curative settings.^114^ Other immunotherapy approaches, such as T cell therapies, also carry a risk of CV toxicity. Chimeric antigen receptor T cell therapy is already used to treat B cell lymphomas with trials ongoing in other tumour types. CV toxicity may occur either as direct toxic events or because of cytokine release syndrome, causing arrhythmias, VTE, myocardial ischaemia and dysfunction.^115 116^

The rapid output of novel therapeutics underscores the importance of robust long-term pharmacovigilance strategies to detect toxicity not seen or underestimated during initial clinical trials, either due to their infrequency, late occurrence or population differences, for example, in performance status or comorbid conditions.^117^

### CV health in cancer survivorship

Treatment-related CV toxicity and the safe delivery of cancer treatment to patients with CVD have been the focus of long-standing collaborations between cardiologists and oncologists ultimately evolving into the subspecialty of cardio-oncology. In addition to acute toxicity, the long-term consequences of cancer treatment on CV health are recognised. This is reflected in the guidelines (table 1) produced by oncological societies including the ESMO and the ASCO which include recommendations for post-treatment survivorship.^118 119^ Survivorship guidelines produced by the National Comprehensive Cancer Network also recommend all cancer survivors receive CV risk assessment, management and counselling.^120^ Whether these guidelines are implemented in practice is less clear and evidence suggests CV risk factors may be underdiagnosed and undertreated in cancer survivors.^121 122^ The ESC has recently released its first consensus guidelines for cardio-oncology, which in addition to guidance on prevention and management of treatment-related CV toxicity, addresses long-term follow-up and chronic CV complications.^81^ Post-treatment CV guidelines are particularly relevant in good prognosis tumours where CV mortality may exceed that of the primary cancer and thus the potential impact of risk-reducing strategies may be greatest.^12^ Furthermore, development of comorbid CVD may impair the quality of life of cancer survivors, which studies of patient priorities suggest is often valued equally to length of life.^123^

**Table 1:** Summary of key recommendations for asymptomatic cancer survivors after completion of anticancer therapy in guidelines produced by the European Society of Cardiologists (ESC), European Society of Medical Oncologists (ESMO) and American Society of Clinical Oncologists (ASCO)^81 118 119^

Recommendation		ESC (2022)^81^	ESMO (2020)^118^	ASCO (2016)^119^
CV risk factor assessment and modification in accordance with standard primary prevention guidelines		Recommended	Recommended	Recommended
Healthy lifestyle advice including healthy dietary habits, body weight and regular exercise		Recommended	Recommended	Recommended
Education of patients regarding recognition of signs and symptoms of CVD		Recommended		
CV surveillance recommendations for asymptomatic cancer survivors after completion of cardiotoxic therapy	CV risk assessment and risk stratification	Annual CV risk assessment including ECG, naturetic peptides, CV risk factor management according to primary prevention guidelines Repeat risk stratification recommended within first 12 months then again at 5 years to organise long-term follow-up	CV risk factor management according to primary prevention guidelines	CV risk factor management according to primary prevention guidelines
	Surveillance recommendations within 12 months of therapy completion	High risk* Recommends echocardiogram and cardiac serum biomarkers at 3 and 12 months after completion of cancer therapy Moderate risk* Consider echocardiogram and cardiac serum biomarkers within 12 months after completion of cancer therapy Low risk* May consider echocardiogram and cardiac serum biomarkers within 12 months of completion of cancer therapy	**Patients at increased risk of cardiac dysfunction** Consider cardiac biomarkers and cardiac imaging at 6–12 months post-treatment	**Patients at increased risk of cardiac dysfunction** Echocardiogram at 6 and 12 months may be performed
	12 months after completion of cardiotoxic cancer therapy	**Very high risk and early high risk**† Consider echocardiogram at years 1, 3 and 5 after completion of cardiotoxic cancer therapy and every 5 years thereafter **Late high risk**† Consider echocardiogram every 5 years **Moderate risk**† May consider echocardiogram every 5 years **Asymptomatic patients after RT >15 Gy MHD** Non-invasive screening for CAD every 5–10 years **Asymptomatic patients with a history of head/neck RT** Consider carotid ultrasound at 5 years post-treatment and then 5–10 years thereafter **Patients with a history of abdomen and pelvic RT presenting with worsening renal function and/or systemic hypertension** Consider renal artery ultrasound	**Patients at increased risk of cardiac dysfunction** Consider cardiac biomarkers and cardiac imaging at 2 years post-treatment and possibly periodically thereafter **Patients with a history of mediastinal RT** Evaluation for valvular heart disease, CAD and ischaemia recommended at 5 years post-treatment then 3–5 years thereafter	

*High risk: High and very-high baseline CV toxicity risk based on HFA-ICOS assessment; receipt of doxorubicin ≥ 250 mg/m^2^, RT > 15 Gy MHD; doxorubicin ≥ 100 mg/m^2^ and RT 5–15 Gy MHD; high risk haematopoietic stem cell transplant patients; moderate or severe cancer therapy related- cardiovascular toxicity during cancer treatment; new CV symptoms or new asymptomatic abnormalities in echocardiography and/or cardiac serum biomarkers at the end of therapy assessment. **Moderate and low risk:** moderate or low risk pretreatment risk category determined using HFA–ICOS baseline CV toxicity risk stratification.^81^

†ESC risk categories for asymptomatic adult cancer survivors defined as **very high risk**: very high baseline CV toxicity risk pretreatment, doxorubicin ≥400 mg/m^2^, RT >25 Gy MHD, RT >15–25 Gy MHD+doxorubicin ≥100 mg/m^2^. **Early high risk** (<5 years after therapy): high baseline CV toxicity risk, symptomatic or asymptomatic moderate to severe cancer therapy-related cardiac dysfunction during treatment, doxorubicin 250–399 mg/m^2^, high-risk haematopoietic stem cell transplantation. **Late high risk**: RT >15–25 Gy MHD, RT 5–15 Gy MHD+doxorubicin ≥100 mg/m^2^, poorly controlled CV risk factors. **Moderate risk**: moderate baseline CV toxicity risk, doxorubicin 100–249 mg/m^2^, RT 5–15 Gy MHD, RT <5 Gy MHD+doxorubicin ≥100 mg/m^2^. **Low risk**: low baseline CV toxicity risk and normal end-of-therapy cardiac assessment, mild cancer therapy-related cardiac dysfunction but recovered by the end of cancer therapy, RT <5 Gy MHD, doxorubicin <100 /m^2^.^81^

CAD, coronary artery disease; CV, cardiovascular; CVD, CV disease; HFA, Heart Failure Association ; ICOS, International Cardio-Oncology Society; MHD, mean RT heart dose; RT, radiotherapy.

Timing of CV prevention strategies is important; pretreatment risk assessment and optimisation may help to reduce treatment-related CV events and a further planned assessment after completion of therapy offers the opportunity to address individualised long-term risk and prevention strategies.^81^ Surveillance with serum cardiac biomarkers and/or cardiac imaging, usually transthoracic echocardiography, aims to detect subclinical toxicity and allow early cardiac intervention. In addition to surveillance during cardiotoxic cancer therapy, guidelines from ESMO, ASCO and the ESC provide recommendations for post-treatment surveillance for asymptomatic cancer survivors who remain at elevated risk of cardiac dysfunction (summarised in table 1). The ESC guidelines also provide recommendations for risk stratification based on clinical and treatment characteristics to guide the level of required surveillance, both in the first year post-treatment and then with repeat risk stratification after 12 months to arrange longer-term follow-up.^81^

Pretreatment CV risk factor assessment and control is a consistent recommendation within guidelines produced by oncology and cardiology societies. Several tools exist for predicting long-term risk of CVD; however, none of these tools take account of the specific risks associated with a cancer diagnosis and treatment. In an attempt to standardise CV risk stratification for high-risk therapies, the Cardio-Oncology Study Group of the Heart Failure Association (HFA) of the ESC, in collaboration with the International Cardio-Oncology Society (ICOS), have developed risk stratification proformas for those planned to undergo treatment with anthracycline chemotherapy, HER-2 targeted therapy, VEGF inhibitors, second and third-generation BCR-ABL inhibitors, multiple myeloma therapies (proteosome inhibitors and immunomodulators) and combination BRAF and MEK inhibitors.^124^ These estimate the risk of treatment-related CV complications by measuring patient and treatment-related factors including the presence of CVD, medical and lifestyle CV risk factors, cardiac biomarkers and previous cardiotoxic anticancer treatment.^124^ Pretreatment risk stratification allows early identification of patients who require further investigations or cardio-oncology input from the outset.^81^ Repeat risk stratification in the 12 months following completion of high-risk anticancer therapy is recommended to organise long-term follow-up.^81^ The HFA-ICOS proformas were developed based on evidence and expert consensus but require validation. Several studies have already provided initial corroboration, but further validation studies are required to inform and refine use of these tools in clinical practice.^125–128^

Several long-term risk assessment tools are available to estimate the 10-year risk of fatal and non-fatal CVD in primary prevention populations, for example, the Systematic Coronary Risk Estimation 2 (SCORE2) and SCORE2-Older Persons (OP) derived and validated in European populations, and QRISK3 in an English population. The ESC guidelines recommended risk stratification using SCORE2 or SCORE2-OP, for patients without a diagnosis of CVD or other high-risk features, prior to certain anticancer therapies which are associated with an increased risk of myocardial infarction, such as ADT, endocrine therapy for breast cancer, fluoropyrimidines and RT where a volume includes the heart.^81^ While these scores are derived from large populations and play an important role in a primary prevention setting, they have not been specifically validated in patients with cancer. A recent cohort study of cancer as a predictor in CV risk scores in 81 420 English cancer survivors, compared with 413 547 non-cancer controls, found adding a 1-year cancer survivorship variable into a QRISK3-based model met the threshold for inclusion in risk derivation for males and females with haematological cancers and males with solid organ cancers, but not females.^129^ These findings support the need for further research into the performance of CV risk assessment tools in cancer survivors, including drivers of elevated CV risk, and evaluation of cancer survivorship status in future CV risk prediction derivations.

Optimisation of medical and lifestyle CV risk factors is recommended both prior to anticancer treatment and in the post-treatment survivorship phase. Several epidemiological studies report associations between elevated baseline CV risk, cancer treatment-related cardiac toxicity and late CV outcomes in cancer survivors.^10 130^ In a study using the CARDIOTOX registry (NCT02039622) of 1324 patients who underwent medium to high-risk cardiotoxic anticancer treatment, a higher baseline CV risk estimated using SCORE was significantly associated with increased rates of cardiotoxicity and all-cause mortality.^131^ Individual risk factors were common with at least one present in 67.5% including a new diagnosis of hypertension, diabetes and hypercholesterolaemia detected in 2.1%, 5.0% and 24.4% of the cohort, respectively, at baseline. A further 13.9%, 10.3% and 9.8% had a subsequent diagnosis of hypertension, dyslipidaemia and diabetes during the 2-year follow-up period.^131^ Risk factor control declined during cancer treatment, despite management in a dedicated cardio-oncology service.^131^ Similarly, studies show declines in adherence to medication for hypertension, dyslipidaemia and diabetes mellitus during cancer treatment, and high levels of non-adherence in cancer survivors are associated with increased CV events.^132 133^ Re-evaluation at the end of cancer treatment offers the opportunity to reassess and optimise risk factors, including evaluation for non-adherence and patient education of the benefits of risk factor control.

The benefits of a healthy lifestyle should be discussed including smoking cessation, weight management, diet and regular physical activity. In the general population, the role of a healthy lifestyle in prevention of diseases including cancer and CVD is well established.^134^ A recent study using the UK biobank found a healthy lifestyle was associated with slower development of CVD and type two diabetes including in cancer survivors with similar levels of risk reduction to those seen in people without cancer.^135^ Furthermore, in a study of 992 stage III colon cancer survivors after a median follow-up of 7 years, higher healthy living scores (comprising BMI, physical activity and diet) were associated with lower overall mortality (HR 0.58; 95% CI: 0.34 to 0.99; p=0.01 for trend) and increased disease-free survival (HR 0.69; 95% CI: 0.45 to 1.06; p=0.03 for trend).^136^ Studies of patient preferences in survivorship demonstrate high levels of interest in health promotion including on nutrition, weight management and exercise.^137^ Recommendations regarding diet, exercise and healthy body weight maintenance are included in numerous post-treatment survivorship guidelines, largely extrapolated from disease prevention guidance.^118–120 138 139^ Diet and physical activity approaches advocated for CVD prevention are also recommended.^81 118 140^ Key recommendations include maintenance of a healthy body weight through nutrient-rich foods including varied fresh fruits, vegetables and whole grains, while limiting intake of refined grains, processed foods, sugar-sweetened beverages, salt, alcohol, processed and red meat.^118–120 136 138 139^ Regular physical activity is also advised, aiming for a minimum of 150–300 min of moderate-intensity physical activity, or 75–150 min of vigorous-intensity physical activity, or an equivalent combination per week; strength training and limiting time spent sedentary.^120 138 139 141^

While patient education and counselling is recommended, eliciting impactful behavioural change remains a challenge. Given the potential benefits, including in addressing lifestyle contributions to health disparity with social deprivation in both CVD and cancer, research and adoption of effective health promotion strategies should be a priority. This is also important given detrimental impacts of cancer treatment on CRF which is a modifiable target and independently associated with survival.^44 49^

Exercised-based interventions have been proposed and are supported by high levels of patient interest, and a growing body of literature supports their safety and benefits.^142–145^ The positive impact of regular physical activity on CV events and mortality is well known, and studies in cancer survivors similarly demonstrate significant reductions in CV events and mortality.^146–148^ Meta-analyses of observational studies have found reduced all-cause and cancer-specific mortality in association with higher levels of physical activity.^149 150^ Meta-analyses of randomised exercise-based interventions also demonstrate benefits to CRF, muscle strength, health-related quality of life, cancer-related fatigue, anxiety and depression.^142 144^ Dedicated cardio-oncology rehabilitation approaches are currently under development.^81 151^ This approach is recommended by the American Heart Association, modelled on cardiac rehabilitation for those with coronary artery disease where it reduces hospital admissions, CV mortality and all-cause hospitalisation, and improves health-related quality of life.^139 151 152^

## Summary and future directions

CV health in long-term cancer survivors is an emerging area of cardio-oncology research (Box 1). Growing evidence points to elevated risk of CVD in cancer survivors which can manifest years after treatment, with studies highlighting the dynamic nature of risk over time and by tumour type. Epidemiological studies, however, often lack detailed patient and treatment characteristics required to characterise key drivers of late risk which are thought to reflect a combination of overlapping aetiologies, pathophysiological processes, and both direct and indirect treatment impacts. The development of survivorship programmes to optimise CV health provides an opportunity to mitigate CV impacts of cancer treatment and improve survival and quality of life in cancer survivors.

Box 1 **Questions for future research**

- What are the most effective approaches to reduce long-term cardiovascular (CV) morbidity and mortality in adult cancer survivors?
- What is the impact of post-treatment cardio-oncology recommendations, including risk prediction and screening, on outcomes in adult cancer survivors?
- How can long-term CV risk prediction be improved in adult cancer survivors?
- How could improved understanding of overlapping pathophysiological processes be used clinically, for example, through development of novel biomarkers or treatment approaches?

Several high-risk treatments are recognised in guidelines, and future studies should aim to validate newly recommended risk stratification tools and assess the impact of post-treatment screening. With the continuing rapid development of novel cancer therapies, approaches to reduce and monitor CV toxicity, and robust pharmacovigilance strategies are important. Consideration of collection of CV outcomes in oncology trials and vice versa with standardisation of CV outcome reporting offers the opportunity to improve understanding of the interplay between these diseases and treatment-related toxicity. Preclinical studies highlight potential interactions of these diseases through several complex pathophysiological processes. As the population ages, improved understanding of the impacts of multimorbidity is more important than ever. Preclinical and translational research into cancer and CVD may provide new insights into novel biomarkers or therapeutic approaches.

Improved long-term CV risk prediction in cancer survivors requires prospective assessment of current primary prevention CV risk prediction tools and recent evidence supports the evaluation of including cancer indices in future score derivations. Risk factor modification is a central component of primary and secondary CV prevention strategies and improving risk factor control in this population requires increased awareness of potential risk among healthcare providers and collaborative approaches between oncologists, primary care and cardio-oncology services. Cardio-oncology prehabilitation and rehabilitation approaches are a possible approach to improving CV risk in certain high-risk cancer survivors, with research into those most likely to benefit and assessment of impact over the longer term required.

### Search strategy

Searches were performed up to February 2023 using PubMed and Medline databases, with various iterations of key search terms “cancer survivors”, “neoplasms”, “cardiovascular disease”, “cardiotoxicity”, “heart disease risk factors” with subheadings and subcategories relating to epidemiology, aetiology, genetics, pathophysiology, survival, cardiovascular risk assessment, risk factor modification, health promotion, quality of life and patient preferences. We also searched relevant clinical guidelines produced by the European Society for Medical Oncologists (ESMO), American Society of Clinical Oncology (ASCO), European Society of Cardiology (ESC), and National Comprehensive Cancer Network. The reference lists of retrieved articles were also searched. Peer -reviewed systematic reviews and meta-analyses, large epidemiological studies and randomised control trials (RCTs) were prioritised where available.

## Data Availability

No data are available.
